# The quest for a metabolic theory of everything

**DOI:** 10.1590/2359-3997000000172

**Published:** 2015-08-24

**Authors:** Fernando M. A. Giuffrida

**Affiliations:** 1 Departamento de Ciências da Vida Universidade do Estado da Bahia Salvador Brasil Departamento de Ciências da Vida, Universidade do Estado da Bahia (UNEB), Salvador, Brasil.; Universidade Federal de São Paulo São Paulo Brasil Programa de Pós-Graduação em Endocrinologia, Disciplina de Endocrinologia, Universidade Federal de São Paulo (UNIFESP), São Paulo, Brasil.; Centro de Diabetes e Endocrinologia do Estado da Bahia Salvador Brasil Centro de Diabetes e Endocrinologia do Estado da Bahia (CEDEBA), Salvador, Brasil

Since the many groundbreaking discoveries of Physics scattered all throughout the twentieth century (and laureate with too many Nobel prizes to enumerate here), Physicists have been painstakingly searching for a theory of everything which could hypothetically unite Quantum Physics and Relativity, in order to explain most phenomena from the minute weird world of particles all the way through the colossal domain of cosmic bodies (1). Physicians are no strangers to this kind of pursuit (2).

In this edition of the *Archives of Endocrinology and Metabolism*, three articles approach different aspects of the Metabolic Syndrome (3-5). The metabolic syndrome (and from now on I deliberately drop the capitals) has probably been one of the most popular philosophical constructs in the history of medicine. It was created by Prof. Gerald Reaven in 1988, in an attempt to unify the pathophysiological role of insulin resistance (IR) in hyperglycemia and cardiovascular risk (6). It has been seen for long as some sort of Holy Grail of metabolic disease, despite intense efforts from its creator to prevent the juggernaut from being unleashed (6-8). A quick PubMed search of the term “metabolic syndrome” yields 39854 entries (9), most of them apparently using the term in its current meaning.

Many problems may arise when applying the diagnosis of the metabolic syndrome to describe the clustering of cardiovascular risk factors (CVRFs). The main four are the following, in the opinion of this author: 1) The existence of multiple patterns of clustering; 2) Applying the concept of metabolic syndrome to diabetes; 3) Overrating the role of IR in the pathogenesis of type 2 diabetes; and 4) The statistical caveats of the interrelation among the metabolic syndrome and its components.

The study by Herrera and cols. (3) approaches the involvement of polymorphisms in genes related to the Renin Angiotensin System, namely *ACE*, *AGT* and *AGTR1*, in the clustering of CVRFs in Chilean individuals. It starts by regarding the existence of the metabolic syndrome as unquestionable, then dividing patients in groups according to its presence, and only thereafter starting to scientifically investigate differences between both groups. It can be considered guilty of committing one specific sin: the style of circular reasoning that analyzes differences regarding components of the metabolic syndrome in subgroups both with and without this diagnosis. Nevertheless, it provides one important argument to lay stress on problem number 1) above. In showing that rs4340 genotypes DD and ID and rs5186 are associated to metabolic syndrome only in women and that rs4340 is associated to waist circumference only in individuals without the syndrome, the paper reinforces the view that CVRFs clusters come in various shapes and sizes, even in the same population. Using or not the term metabolic syndrome does not invalidate the findings, though, since they could simply be put in other words: the described polymorphisms are associated with number and magnitude of CVRFs (notwithstanding genomic significance and replication, which we shall not delve into here).

The paper by Madani and cols. (4) analyzes 624 women with polycystic ovaries syndrome. Again, patients are divided in groups with and without the metabolic syndrome, and only afterwards the investigation of scientific hypotheses begins. The same circular reasoning occurs here. The study finds that the prevalence of metabolic syndrome components is greater in women with the syndrome, and also that the latter is more common in obese individuals. Around 20% of the sample has prediabetes or diabetes. Clustering of CVRFs could possibly be different between this group and the remaining normoglycemic 80%, but this was not directly approached.

The paper by Melo and cols. (5) wisely calls the clustering of cardiometabolic risk factors no other additional names. A sample of Brazilian children and adolescents from 11 to 19 years old was assessed. It addresses the clustering of CVRFs and the inception of cardiovascular risk still in childhood. This article provides some previously known information, but adds to the body of numerous fine examples showing that there are several patterns of clustering, accounting for several hypothetical types of metabolic syndromes. Although beyond the scope of the study, the discussion states that studying the clustering of CVRF only makes sense when connected to the outcomes. The article brings one practical consequence for the relationship between hyperglycemia and other cardiovascular risk factors, based on the finding of glucose changes being the least prevalent CVRFs in the sample. This makes the phenomenon of progressive beta-cell failure, a necessary condition for the development of type 2 diabetes, more detached from the CVRFs related to IR.

The three papers above are in themselves a collective example of the existence of multiple patterns of CVRF clustering. If we embarked on a mental exercise of considering those the only evidence about CVRF clustering in literature, we most likely would not be able conclude that this cluster is a single pathophysiological entity. Population differences among the three studied samples are almost certainly substantial, although they cannot be scientifically evaluated here. One could argue that this very statement demonstrates multiple patterns of CVRF clustering. Reaven has criticized the utilization of multiple criteria for the metabolic syndrome as early as 2004 (7) and later questioned himself if this diagnosis was at all necessary (6).

This brings us to the second problem, of applying the metabolic syndrome to individuals with diabetes. It can be seen in Brazilian individuals that clustering of metabolic abnormalities seems to be related to the degree of hyperglycemia in both type 1 (10) and type 2 diabetes (11). This could suggest that beta-cell dysfunction is responsible for part of the clustering, undermining the putative central role of IR.

This, in turn, relates directly to overrating the role of IR in the pathogenesis of type 2 diabetes. In these days of DeFronzo’s ever growing Ominous Octet (12) and following the explosion of genetic data linking type 2 diabetes to insulin secretion rather than IR seen from 2007 on (13), we cannot deny that IR is an important CVRF and that it is associated with type 2 diabetes, but the superposition among individuals with and without diabetes regarding IR is too overwhelming to be overlooked (8).

The metabolic syndrome is also a statistical conundrum. It might seem straightforward to statistically test the syndrome along with its components, but this is not easily accomplished with most popular multivariable methods such as regression or general linear models. The papers by Herrera and cols. and Madani and cols. are just two of numerous examples of studying differences among components of the syndrome in individuals both with and without the syndrome itself (3,4). Multicollinearity among variables makes it surprisingly difficult to insert all the components of the syndrome in multivariate statistical models (10).

Supporters (14) and detractors (15) of the metabolic syndrome have defended their points of view with much passion in the last decades, but none other than the creator of the syndrome has attempted to discredit (or at least better explain) his initial proposition, firstly pointing out the problem of multiple patterns of clustering (7), then questioning the necessity of this diagnosis (6), and finally in 2011 appealing to everyone for cutting short the metabolic syndrome game (8). One letter to this journal, commenting on the now famous merry-go-round editorial by Prof. Reaven, has optimistically stated that the creator had killed the creature (16).

Nevertheless, a more precise and up-to-date metaphorical description of the metabolic syndrome would be a shape-shifting and scheming creature more akin to Mr. Edward Hyde (or maybe its green-skinned pop culture counterpart). A PubMed search for the term “metabolic syndrome” limited to the present year of 2016 yields 2173 entries (17). Still a staggering average of 16 articles a day! The famous Mark Twain misquote seems tailor-made for the current situation of the metabolic syndrome: the reports about its demise have in fact been greatly exaggerated.
